# Medical and Philosophical Intelligence

**Published:** 1817-10

**Authors:** 


### MEDICAL AND PHILOSOPHICAL INTELLIGENCE.

### *Analysis of the Labours of the Royal Academy of Sciences of the Institute of France during the Year* 1810.

*Physical Part;* by M. LE CHEV. CUVIER, Perpetual Secretary.

BOTANY *and Vegetable Physics.*—One of the most important botanical considerations, and which connects it more than any other branch of natural history with the physical sciences in general, is vegetable geography, or the science of the laws of the distribution of plants according to the height of the pole, the elevation of the soil, the temperature, and the dryness or moisture of the climate.

M. de Humboldt, whose travels have advanced so remarkably this branch of knowledge, as well as several others, has just published a kind of complete treatise of it, under the title of Prolegomena de Distributione Geographica Plantarum secundum Cœli Temperiem et Altitudine Montium, a work in which he gives at the same time profound researches on the distribution of heat, whether relative to the position of places, or to the seasons of the year. For not only the lines under which the mean annual temperature is the same are far from being parallel to the equator; but the places which have their whole mean heat equal are far from having their summers and winters similar. This mean heat may be more or less unequally spread through the whole of the year, and it is obvious that all these differences ought to have considerable influence on the propagation of plants. The author then passes to the differences which result from the elevations, which vary considerably, and follow different laws in different places. Finally, M. de Humboldt comes to a consideration quite new, on which he had likewise published a dissertation in French; viz. that of the distribution of vegetable forms. On comparing in each country the number of plants of certain well-determined families with the whole number of vegetables, we discover numerical ratios of a striking regularity. Certain forms become more common as we advance towards the pole; while others, on the contrary, augment towards the equator. Others acquire their maximum in the temperate zones, and diminish equally by too much heat and cold; and, what is remarkable, this distribution remains the same round the whole globe, following not the geographical parallels, but those which M. de Humboldt calls *isothermic;* that is, lines of the same mean temperature. These laws are so constant, that if we know in a country the number of species of one of the families, of which M. de H. has given a table, we may nearly conclude from it the total number of plants, and that of the species of each of the other families.

Even at present, M. de Humboldt is publishing in London, along with Mr. Horner, a quarto volume, which will exhibit 200 species of mosses, lichens, and other cryptogamous plants. He has presented one of the plates to the Academy.

M. de Beauvois, whose perseverance is equally deserving of praise in publishing the plants and insects collected in his travels, has given this this year the fourteenth and fifteenth parts of his Flora of Owara and Benin; and, not satisfied with these ancient collections, he has taken advantage of the remarkable and disagreeable wetness of this year to prosecute the study of the fungous family of plants. The constant rains brought so many of them forward, that several presented themselves which had escaped preceding botanists, even the most successful in that kind of study. For example, a variety of sclerotium, which reduced the crop of kidney beans without branches to nearly a third of the usual quantity; a new species of spheria, which injured the onions very much; a new species of uredo, which was still more pernicious to them; and, what is remarkable, and offers few examples in the vegetable kingdom, a new species of parasitical plant, which grows upon another parasitical plant, and injures the vegetable considerably, which is obliged to nourish them both. It is a species of tubercle, which fixes itself above the root of the orobanche racemosa, which is known to grow parasitically upon hemp. This tubercle possesses characters which makes it approach to trufles and to sclerotium; but with distinctions, which constitute it a new and intermediate genus. As M. de Beauvois proposes next year to repeat his observations on this very remarkable plant, he has deferred assigning it a name till he has more accurately determined its manner of growing, and all the details of its organization.

We announced last year the opinion of M. de Candolle respecting that injurious substance called ergot (the *spur)*, and which shows itself upon the spike of rye, and of some other corns, especially in moist countries and seasons. The year 1816, unfortunately, produced a great deal of it; and M. Virey has made some researches on it, which lead him to consider the *ergot*, according to the old opinion, as a degeneracy in the grain, and not as a fungus of the genus sclerotium, as is the opinion of M. de Candolle. He says that he has observed grains infected with the *spur*, which not only preserved their natural form, but which still continued to display the remains of stigmata; and he mentions the assertion of M. Tessier, that we often observe grains one half of which only is infected, and sometimes the half towards the summit, sometimes towards the base.

M. Vauquelin has made a comparative analysis of healthy rye, infected rye, and of a sclerotium well defined as such.

In the infected grain we find neither starch nor gluten in their natural state, though it contains a mucous matter, and abundance of a vegeto-animal matter disposed to putrefaction. It contains a fixed oil quite formed. The principles of sclerotium are very different. These experiments, without being decisive, have induced some persons, as well as M. Virey, to hesitate whether the *ergot* be a fungus.

[We cannot help repeating our wish for some information on this subject from our botanical correspondents.]

*Zoology, Anatomy, and Animal Physiology.*—Animals have likewise their geography; for nature confines each species within certain limits, by ties more or less analogous to those which confine vegetables. Zimmerman formerly published a work on the distribution of animals, which was not destitute of celebrity. Latreille has has just published one on the distribution of insects. It is obvious that it must be intimately connected with that of plants; and in reality we find, upon the mountains of a warm country, the insects that inhabit the plains of colder countries. The difference of 10 or 12 degrees of latitude occasions always at an equal height particular insects; and, when the difference amounts to 20 or 24 degrees, almost all the insects are different. We observe analogous changes corresponding to the longitude, but at distances much more considerable.

All Paris had it in its power to see a woman brought from the Cape of Good Hope distinguished by the name of the Hottentot Venus. She belongs to a nation in the interior of Africa celebrated among the colonists at the Cape for its ferocity, and which the barrenness of the country that they inhabit, and the persecutions of the people in their neighbourhood, contribute equally to render miserable. The smallness of their size, the peculiar shape of their heads, the yellowness of their skin, and particularly the enormous size of the hips in the women, seem to render them a very distinct race from the negroes and caffrees by whom they are surrounded. A great deal has been said about the apron of these women, which the first travellers described very inaccurately, while recent voyagers have gone so far as to deny its existence altogether.

The woman of whom we have spoken having died in Paris, M. Cuvier had an opportunity of dissecting her, and of determining the peculiarities of her structure. She possessed the apron; but it was neither a fold of the skin of the belly, nor a peculiar organ. It was only a considerable prolongation of the upper part of the nymphæ, which fell over the opening of the vulva, and covered it entirely. The protuberance of the hips is composed of a cellular matter, filled with fat, nearly similar to the bunch on the back of camels and dromedaries. The skeleton preserves no traces of it, unless it be a somewhat greater size of the edges of the pelvis. The head exhibited a singular mixture of the characters of the negro and of those of the Calmuc. Finally, the bones of the arm, remarkable for their smallness, exhibit some distant relations with those of certain apes.

One of the most formidable serpents after the rattlesnake is the yellow viper, or *fer-de-lance*, of Martinique and St. Lucia, on which M. Moreau de Jonnés has read to the Academy an interesting Memoir. Naturalists at present place it among the trigonocephali, characterized by the pit situated behind the nostrils. It infests the principal of the colonies that remain to us. Some affirm that it was formerly brought out of hatred to the Carabees by the Arrouages, a small tribe on the borders of the Oronoko—a tradition which might explain why it has remained unknown in the other Antilles. From the sea-shore to the top of the Mornes we are exposed to its attacks; but its principal refuge is among the sugar-canes, where multitudes of rats serve it for food, and where it is propagated with a rapidity proportional to the number of its young, which amounts to 50 or 60 at a time. Its length is sometimes more than six feet. Vain attempts have been hitherto made to destroy these these vipers by pursuing them with English terriers. M. Jonnès proposes to try against them that bird of prey with long legs called *messager* or *secretaire (falco serpentarius*, L.), which devours so many serpents in the neighbourhood of the Cape of Good Hope; and the administration has already thought of transporting this useful species to Martinique. Probably the *mangouste* would not render less important services.—*Annals of Philosophy.*

---

### *City of London Truss Society, for the Relief of the Ruptured Poor.*

| | | |
|---|---|---|
| Patients relieved by this Society in | 1808 | 227 |
| | 1809 | 570 |
| | 1810 | 813 |
| | 1811 | 1094 |
| | 1812 | 1606 |
| | 1813 | 1800 |
| | 1814 | 2064 |
| | 1815 | 2473 |
| | 1816 | 2610 |
| First Six Months of | 1817 | 1518 |
| | | 14,835 |

The following statement of the situation and occurrence of hernia, at different periods of life, has been extracted from the register of the patients relieved by the City of London Truss Society, within the short period of nine years and six months.

In 13661 cases, 11,192 were males, and 2469 were females.

| Males. | Females. | | |
|---|---|---|---|
| 2199 | 44 left inguinal | 6237 inguinal | 7189 single |
| 2949 | 45 right inguinal | | |
| 69 | 395 left femoral | 952 femoral | |
| 77 | 411 right femoral | | |
| 4487 | 37 double inguinal | | 4860 double |
| 65 | 271 double femoral | | |
| 56 | 542 umbilical | | 703 |
| 32 | 73 ventral | | |
| | 2 perineal | | 2 |
| | 2 obturator | | 2 |
| 18 | 30 have undergone operations—all except *one* have been completely successful | | 48 |
| 145 | 132 with umbilical and inguinal hernia have been cured | | 277 |
| 89 | 21 with prolapsus ani | | 110 |
| | 462 with prolapsus uteri | | 462 |
| 6 | 2 with varix of the abdominal veins | | 8 |
| 11,192 | 2469——13,661 | | 13,661 |
| | | | 1012 patients. |

| | | | | | | |
|---|---|---|---|---|---|---|
| 1012 | patients were relieved with trusses under 10 years of age. | | | | | |
| 685 | ditto | between | 10 | and | 20 | ditto. |
| 1367 | ditto | ——— | 20 | and | 30 | ditto. |
| 2244 | ditto | ——— | 30 | and | 40 | ditto. |
| 2430 | ditto | ——— | 40 | and | 50 | ditto. |
| 2382 | ditto | ——— | 50 | and | 60 | ditto. |
| 1648 | ditto | ——— | 60 | and | 70 | ditto. |
| 544 | ditto | ——— | 70 | and | 80 | ditto. |
| 72 | ditto | ——— | 80 | and | 90 | ditto. |
| 4 | ditto | ——— | 90 | and | 100 | ditto. |
| 12,388 | | | | | | |

Eight females each had umbilical and double femoral hernia, 2 females had large ventral and double femoral hernia, 6 females had umbilical and ventral hernia, 1 female had right femoral hernia and prolapsus uteri, 1 female had ventral hernia and prolapsus uteri, 1 female had umbilical hernia and prolapsus uteri, 6 females had umbilical and single femoral hernia, 4 females had right inguinal congenital hernia, 7 females had left inguinal and right femoral hernia, 1 female had left femoral and right obturator hernia, 5 females had ventral and single femoral hernia, 1 female had double femoral and right inguinal hernia, 6 females had double inguinal and umbilical hernia, 1 female had right inguinal and umbilical hernia, 1 female had right inguinal and ventral hernia, 7 females had left inguinal and left femoral hernia, 1 female had left femoral hernia outside of the femoral vessels, 1 female had right femoral hernia outside of the femoral vessels.

Thirty males each had left femoral and right inguinal hernia, 9 males had left inguinal and left femoral hernia, 21 males had left inguinal and right femoral hernia, 10 males had double inguinal and right femoral hernia, 8 males had double inguinal and left femoral hernia, 6 males had right inguinal and right femoral hernia, 2 males had double inguinal and double femoral hernia, 6 males had double femoral and right inguinal hernia, 3 males had ventral and right inguinal hernia, 1 male had ventral and right femoral hernia, 2 males had ventral and left inguinal hernia, 2 males had ventral and double inguinal hernia, 1 male had double ventral and double inguinal hernia; 1 male had left inguinal, umbilical, and ventral hernia; 1 male had double inguinal, umbilical, and ventral hernia; 1 male had umbilical and left inguinal hernia, 3 males had umbilical and right inguinal hernia, 1 male had umbilical and left femoral hernia, 1 male had umbilical and right femoral hernia, 10 males had umbilical and double inguinal hernia, 2 males had double femoral and right inguinal hernia, 1 male had right femoral hernia outside of the femoral vessels, 1 male had double inguinal hernia and prolapsus ani.

Six hundred and ninety-eight patients had congenital hernia.

*Fœtus with a Double Head and Heart.* (Giorn della Soc. Medic. Chirurg. di Parma, vol. xv.)—One of the last observations of the illustrious anatomist Paul Mascagni, whom Italy and the learned world have just lost, is relative to a male fetus, double in the upper parts and united in the lower. One only pericardium contained two hearts: there were double lungs, two aortal arteries, two upper *venæ cavæ*, but only one lower, which, after having traversed the diaphragm, divided itself into two branches, one of which turned to the right and the other to the left. There existed two thoracic ducts, each following a vertebral column, between the azygos vein and the aorta, going, as usual, into the angle of the subclavian veins and the internal jugular. There was but one liver, but two spleens, two stomachs with their intestines, which, towards the colon, united themselves in one canal. This singular fetus had two heads and two vertebral columns, well formed in all respects: we remarked the vessels and nerves of a size proportionate to the parts which they were to furnish with blood. This fœtus was born in the province of Pilago.

After having remarked what there is of singularity in the order which nature follows even in her aberrations, as if it was not permitted her to exceed certain limits even in her deviations, it must be added, that it is less astonishing than we shall at first be disposed to believe, to find this regular arrangement in monsters. All those who survive are curious subjects of observation, and will, perhaps, one day assist in unveiling the physiological mechanism of the formation of living beings.

---

*Memoir on the Operation of the Cataract.*—M. PHILIBERT ROUX, Second Surgeon of the Hospital of the Charity, recently read, at the Academy of Sciences, a Memoir and Observations on the Operation of the Cataract by Extraction. The Academy has nominated Messrs. Percy and Deschamps to report upon it. M. Roux, from his experience, having operated on upwards of 600 patients, prefers this method to that of *abaissement* [depressing or couching]. We will not anticipate the judgment of the Commissioners, but simply observe, that, from the statement of M. Roux, it appears that four-fifths of those operated were cured, while by abaissement the proportion does not exceed two-thirds.

In a subject which seemed exhausted, M. Roux has been able to make observations which had escaped all his predecessors: for example, he has perceived that, though the motions of the iris are more lively in eyes which have cataracts than in those in health, these motions are afterwards entirely and for ever destroyed in most of the patients operated on by either of the two methods. He thinks that this fixity of the iris is owing to the adherence contracted between this membrane with the capsule of the vitreous humour or the cristalline membrane. He has discovered, too, that matters being equal in other respects, light eyes offer greater chances of success than dark ones, a singularity which he compares with the known known fact that we more frequently observe on dark small sunken eyes, than on light-coloured prominent ones, cataracts of a bad nature, and especially cataracts complicated with amaurosis or paralysis; and for which we ought to abstain from all operations whatever.

---

Of all the forms in which Digitalis may be given, Dr. HOHNBAUM observes the tincture to deserve the preference, the powder and the decoction acting particularly on the stomach and the intestinal canal, and less upon the vascular and lymphatic system. Besides this, the tincture has the advantage that the stomach and intestines bear this preparation longest, provided it be properly prepared. He thus recommends the following formula:

℞. Herb. digital. purpureæ concis. ℥ij. Spirit. vini rectificat. Aq. Cinnamom. simpl. ad ℥iij. M. digere per quatriduum in loco calido. Tincturam leniter expressam filtra et serva usui.

This preparation is less irritating than other spirituous tinctures; and Dr. H. uses it in hydropical and phthysical disorders, in affections of the vascular and lymphatic systems, founded on a high degree of irritability and excitability, but more particularly in hydrothorax, proceeding from catarrhous and inflammatory affections of the lungs, and the mucous glands of the bronchiæ, connected with an inflammatory diathesis. The dose given by Dr. H. is from 15 to 25 drops every five or six hours.

---

*List of Patients admitted into all the Civil Hospitals of Paris, from the 1st to the 30th of July,* 1817, *both inclusive.*

| | |
|---|---|
| Uncharacterised fevers | 27 |
| Intermittent ditto, various | 206 |
| Bilious or gastric ditto | 243 |
| Adynamic or putrid ditto | 10 |
| Catarrhal ditto | 9 |
| Phlegmasiæ, internal and external | 80 |
| Ophthalmia | 23 |
| Rheumatism | 32 |
| Diarrhœa and dysentery | 13 |
| Erysipelas | 13 |
| Phlegmasiæ of the organs of respiration | 79 |
| Consumption | 36 |
| Apoplexy and recent paralysis | 16 |
| Dropsy and anasarca | 43 |
| Small-pox | 14 |
| Metallic colics | 10 |
| Sporadic and chronic disorders, and the result of accidents | 218 |
| Itch | 79 |
| Total | 1151 |

***List of Patients admitted into all the Civil Hospitals of Paris, from the 1st to the 31st August, 1817, both inclusive.***

| *From August 1st to* | *10th* | *20th* | *31st* |
|---|---|---|---|
| Uncharacterised fevers | 11 | 12 | 5 |
| Intermittent ditto, various | 69 | 63 | 64 |
| Bilious or gastric ditto | 61 | 72 | 67 |
| Adynamic or putrid ditto | 6 | 18 | 8 |
| Catarrhal ditto | 0 | 0 | 1 |
| Phlegmasiæ, internal and external | 46 | 26 | 25 |
| Ophthalmia | 9 | 7 | 4 |
| Rheumatic pains | 14 | 14 | 11 |
| Diarrhœa and dysentery | 3 | 5 | 7 |
| Erysipelas | 0 | 4 | 4 |
| Phlegmasiæ of the organs of respiration | 0 | 30 | 29 |
| Phthisis | 10 | 12 | 17 |
| Dropsy and anasarca | 14 | 19 | 10 |
| Apoplexy and recent paralysis | 0 | 3 | 4 |
| Variolous | 1 | 7 | 6 |
| Metallic colics | 5 | 4 | 3 |
| Sporadic and chronic disorders, and accidents | 56 | 76 | 60 |
| Itch | 27 | 35 | 20 |
| | 332 | 407 | 345 |

***Meteorological Report.***

| *From July 1st to* | *10th* | *20th* | *30th* |
|---|---|---|---|
| Maximum of barometer | 28.2 $\frac{7}{12}$ | 28.2$\frac{1}{12}$ | 28.3$\frac{7}{12}$ |
| Minimum of ditto | 27.11$\frac{6}{12}$ | 27.7$\frac{2}{12}$ | 28.0$\frac{4}{12}$ |
| (centigrade) | | | |
| Max. of thermometer | 20° | 19° | 21°.9 |
| Min. of ditto | 12°.9 | 10° | 10° |
| Max. of hygrometer | 97°.2 | 98° | 91° |
| Min. of ditto | 84° | 87° | 87°. |

CHEVALLIER, Optical-Engineer.

***Reigning Disorders;*** by Dr. DE MONTEGRE.

*From the 1st to the 10th July.*—These ten days have been very rainy. The most common affections have been sudden colic, with violent griping, sometimes accompanied with serous diarrhœa, and very abundant, producing sudden and extreme weakness: the pains principally affected the hollow part of the stomach, or more accurately the line of the transverse arc of the colon. In general, the sensibility of the abdomen was augmented, but not to the degree of inflammation; the pulse lively, hard, and very chordy; with all the consecutive symptoms of violent diarrhœa. The treatment which has been attended with the quickest success is emollients, emulsions with a slight portion of nitre; draughts in which a little syrup of ipecacuanha is mixed with double the quantity of syrup of dioscordium, diluted in water slightly spirituous; clysters with a decoction of the roots of althæa officinalis and poppy heads. I once employed ployed the decoction of the bark of *quassia simaruba* twice or thrice in the course of the day. The patients submitted to this treatment were speedily relieved from an indisposition attended with such alarming symptoms.

*From the* 10*th to the* 20*th.*—The season is completely deranged: the wind, which from the commencement of the year had been continually northerly, has turned suddenly to the west and south: there are no longer threatening, but passing, storms: they have given way to abundant rains, floods, and torrents, which succeed each other almost without intermission. Gastric affections of all kinds have been very common, either as in the first decade of this month, or attended with febrile symptoms. It would be a curious object of research to seek the modifications of the human constitution, whereby persons subjected to the same influence experience effects so different. We possess but slight and general data on this subject, which merits the highest consideration. I have used emetics with success. I thus prepare them, whereby the most delicate persons may take them without the nausea they generally create:—Take one drachm of ipecacuanha, bruised or in powder—boil it a quarter of an hour in a pint of water—filter the liquor through a linen cloth, and sweeten it with sugar or simple syrup. It is to be administered in three or four doses, by which means the vomiting can be carried to the point desired according to the case, or the state of the patient. Aromatics should not be united with the emetic, not even to disguise the taste.

*From the* 20*th to the* 30*th.*—The weather has greatly improved, and promises a delightful season for the harvest. The most numerous disorders amongst those admitted into the hospitals are bilious and gastric fevers; but, within these few days, several cases have come before me which required sanguine evacuations, and that in persons of an advanced age, or in indigence, viz. cephalalgy, giddiness, and vertigo. All these symptoms increased with the least fatigue, and particularly when the patients went up a staircase. Six, eight, to ten leeches, applied opportunely, have generally removed these symptoms.

---

*Extract of a Letter from* M. DE VARENNES, Mayor of the Town of Coulomiers, *to* Professor CHAUSSIER, *on an unmarried Woman who, for nearly Eleven Years, had taken no solid Food.* Communicated to the Faculty of Medicine of Paris.

In 1783, when I was in garrison at Aire, I heard of a girl called Marie-Joseph Dahl, native of the village of Disonguin, at a short league from the town, who, for several years, had taken no solid food. As the case appeared to me very extraordinary, I wished to ascertain it myself, and collect all the details. I went and found this unfortunate woman, then of the age of 43. She was lying, or rather sitting, huddled up on a bed of straw, her head resting on her knees—a large coarse towel served her for a sheet, and another to cover her, for she had no clothes on—her skin was tolerably white, white, but she was nothing, as it were, but skin and bone. I was assured that for ten years this unfortunate being had been in that motionless state; that she was physically and morally insensible; and that, during the whole time, she had taken no other nourishment than water, slightly sweetened with honey, which is called in that country *petit lait* (small milk). At the period when I visited her, a few drops were given her twice a-day, but if it was omitted she did not appear to testify any want of it. I was told that for two years she had never changed her position of herself, and had given no signs of life but by an almost insensible respiration, and the movement of the deglutition; and that, if one sought to extend the arm from the body, or to open the knees, as much resistance was felt as if they were branches of trees. The permanence of this state was attested by several persons of credit in the neighbourhood, and particularly by the Curé, who appeared to me a man of learning and sound sense. I visited her myself thrice at intervals of three days, to examine whether any change took place, but I did not perceive any.

This state was the effect of over fatigue and love. She had been servant to a farmer—she loved, and was beloved by, the farmer's son. The father would not consent to the marriage, because the girl was poor. However, one day, during harvest, he said to her in a jest, "Mary, if, in three days, you will reap this field of wheat, without the assistance of any one, I will give you my son." The poor girl believed him, set to, laboured night and day, finished her task, and fell immediately into this deplorable state.

When I visited her, they gave her about two spoonfuls of beverage per diem, a portion of which was lost in administering it. I observed that a few minutes after she had taken it, her countenance was a little flushed. Sometimes she evacuated, in the ordinary way, a yellow matter, not so liquid as the nourishment she took; and, as the jaws were locked, they had broken three teeth, in order to administer refreshment; but, in this operation, as well as in the application of blisters and cupping, and other means that had been employed, she never shewed any sign of sensibility.

In my three visits I was accompanied by M. Gilet, Surgeon-Major of the Regiment. This unfortunate woman died in 1784, one year after my visits, and about eleven after the commencement of the disorder which brought on this immobility of body and long abstinence.

---

*Three Pathological Cases—I. The Vagina reversed. II. Cancer of the right Loin. III. Singular Conformation of the right Auricle of the Heart.* Presented by M. Beclard, Chief of the Anatomical Operations, and Julius Cloquet, Prosecteur.

*I. Vagina reversed, found in a Woman, æt.* 80.—The vagina, thus reversed, formed a long cylindrical tumour, pretty firm, with a large base, and tuberculous at its summit; of a pink colour, with violet spots. The surface of the tumour was covered with transverse

 wrinkles.

wrinkles. The length was three inches towards its summit: it presented two irregular tubercles, separated by a light excavation, corresponding with the vaginal orifice of the neck of the uterus, which was obliterated. The tumour was covered externally by a sort of epidermis, of a peculiar nature: it was a semi-opake pellicle, whitish, pretty strong, with white thicker patches here and there in an irregular manner. The external face of this pellicle was smooth and polished; its internal face, on the contrary, was *villous*, and presented a great quantity of little *papillæ* (protuberances resembling nipples), and little cavities connected with similar inequalities, distributed on the surface of the vagina: in short, the epidermis externally very much resembled the pellicle of old scars or cicatrices, and its inside offered some analogy to the epidermis covering the tongue, and which is easily detached by the action of boiling water.

The base of the tumour presented, on the upper side, the exterior orifice of the canal of the urethra, which was dilated.

Seen internally, the vagina formed a bag, containing, from front to back, 1, the bottom of the bladder, and a part of the *trigone vesical;* 2, the neck of the uterus, and behind it a large end of the peritoneum, which separates it from the rectum; 3, a prolongation of the rectum. A section made at the base of the tumour would have opened the bladder, the neck of the uterus, the cavity of the peritoneum, and the rectum.

The bladder, drawn by the vagina, was prolonged about two inches into the front side of the upper part of the bag it formed. The canal of the urethra had totally changed its direction: it was vertical and straight, so that its external orifice was highest, and the internal the lowest. Also, to probe the bladder, it was necessary to introduce the catheter first backwards and downwards, then vertically and downwards. The *trigone vesical* was found reversed: its position was downwards and backwards, which is the opposite of its ordinary state. The angle of the trigone corresponding with the ureter was higher, and posterior to the other two angles, which are at the orifice of the urethra. The bottom of the bladder stretched beyond the trigone, to form nearly the whole of the prolongation that this organ sent into the vagina.

The ureters passed on the sides of the bladder, and stretched obliquely, as ordinarily happens with the tunics of this reservoir. The bladder presented no alteration in its organization: it contained two roundish calculi, yellowish and furrowed, each of the size of a small walnut, and formed of layers, internally of a fawn colour, the outer layers white.

In pressing the bladder a little, the two calculi were forced into the prolongation of the bladder to the vagina, and it was easy to feel it in the fore and upper part of the base of the tumour. The bottom of the peritoneum, situated between the bladder and the matrix, was shallow, and did not extend into the tumour, like that between the matrix and the rectum.

The matrix was small, flat, lengthened, and soft; it presented a fibrous fibrous tumour of the size of a filbert, towards the lower part it was two inches long, and was turned with the neck, which was very small and remarkable for its length, being four inches long; so that the uterus and its neck measured six inches; the cavity of the neck was free, filled with mucous, and pretty narrow at the lower part; it terminated by a dilatation full of sinuosities, which presented no communication with the exterior, and of which the coats were provided with whitish fibrous columns; the matrix, on account of the length of its neck, was out of the bag which was formed by the inverted vagina.

Behind the neck of the uterus, the peritoneum formed the long bag *(cul de sac)* we have mentioned, and which was prolonged to the summit of the tumour.

The rectum was dilated at its lower extremity, it terminated behind, in the anus; and in front, it sent a prolongation in form of a bag *(cul de sac)*, which passed over the perinæum, and terminated in the posterior and lower part of the bag formed by the vagina; in front it was separated from the neck of the uterus by the peritoneum.

The tumour could be reduced, and then the parts resumed nearly their natural position.

In this case the matrix did not communicate with the exterior on account of the obliteration of its neck.

*II. Cancer of the Right Loin.*—On the body of a man aged 50, we found, in the right lumbar region, a tumour of the size of a child's head, and which had deranged the position of the stomach and the duodenum; this tumour might be felt through the coats of the abdomen, its upper part adhered to the liver by the means of cellular frenulæ; it was very soft, yielded to pressure, and evinced a fluctuation. We found in the posterior part, vestiges of the loin which were flabby and discoloured; the *canaliculus renum* was dilated and strongly injected, and contained flakes of a cancerous yellowish matter, floating in a purulent fluid of a faint smell. This fluid and several of these flakes occupied the cavity of the bladder, though the ureter did not present any sensible dilatation, the tumour was formed of a cancerous cerebriform coat, in which were little cavities filled with blood and pus.

The liver was very soft and presented a great quantity of cancerous tubercles.

The left loin was perfectly sound, as well as the other viscera of the abdomen.

*III. Irregular Conformation of the Heart.*—We present to the society, the heart of a malefactor, æt. 26, and which exhibits the following conformation:—The upper vena cava offers two distinct trunks, which open separately into the cavity of the right auricle; one of these trunks, of which the direction is vertical, and which occupies the ordinary place of the vena cava, only receives the right subclavian vein and the internal and external jugular of the same side, and opens in the upper part of the right auricle; this trunk is not large, the other, which is larger, receives the left subclavian vein, the jugulars of the same side, the lower thyroideans, the great left intercostal costal vein, and several other branches. This trunk is at first horizontal, then becomes vertical in passing before the cross of the aorta with the left division of the pulmonary artery; it next passes above the appendage to the left auricle at the posterior part of this auricle, where it becomes again horizontal, and terminates in the left part of the right auricle by a wide sloping aperture deprived of valves; this aperture is situate at the left of the lower vena cava, and behind the oval space which separates it from the opening of the upper vena cava, so that these two veins open into the auricle at the distance of one inch from each other. The cardiac veins open by three distinct orifices, and deprived of valves in the horizontal portion of the second trunk behind the left auricle, and not, as is ordinarily the case, at the very cavity of the auricle by a single aperture. The heart of this individual offered no other vice of conformation.

---

*Death of* Dr. VALLI.—Dr. Valli, who, during his long residence at Smyrna and Constantinople, believed, from the experiments he had made, that it was possible to prevent the contagion of the plague, embarked a few months ago for the West Indies with the intention of making analogous observations on the yellow fever. Arrived at Jamaica, and, finding no epidemic disorder there, he went to the Havannah, where he was told it existed; again deceived, and resolved to hunt it out wherever it might be, he learnt that it made its ravages in another part of the Isle of Cuba, where it was forbidden to penetrate; inspired by enthusiasm, and sure of his talisman, he resolved to evade the guard stationed round the village where it spread its havoc; he succeeded, and established himself in a house where two individuals had died of it, and a third was already attacked; he carried his temerity so far as to sleep in their bed, but the third day after his passing the line of contagion, he fell a victim to his erroneous opinion of the non-existence of contagion in the yellow fever [we should rather say to his erroneous theory of contagion]. This event is the more to be regretted, as it proves nothing, and as Dr. Valli was well known to all Europe for his talents and intrepidity.

*** At the sittings of the French Institute on Monday the 31st of March, a long memoir was read on Contagion, from which the author drew, amongst others, the following conclusions: speaking of infected air and the insalubrity of the marshes of Italy, that the most dangerous times for breathing infected air is at sun-set and at sun-rise, or a little before it; that combustion destroys or neutralizes its effects, as the labourers who were compelled to breath it preserved themselves from its pernicious effects by keeping up large fires; that its action is most powerful in an empty stomach, breathing it after a hearty meal producing no ill effects; that its transmission might be arrested in a variety of ways, as by trees, which attract or receive and retain its particles, while the unchanged air passes freely; that canvas, muslin, silk, or even gauze, between the current of air and the object, was generally found to be a sufficient protection. From which it appears that the miasma or infectious particles of matter, whichever they be considered, forming contagion, are arrested by any object in the line of the current. We shall develope this important theory in a future Number.

---

*Case of Wounded Bladder, terminating favourably.* By J. DOUGLAS, Surgeon, Hawick.

Captain S. was wounded at the battle of Chippawa, in Upper Canada, on the 5th of July, 1814. The ball, having penetrated the left groin, over Poupart's ligament, by the side of the large blood-vessels of the thigh, fractured the anterior part of the pelvis, passed through the bladder of urine, slanting obliquely across the basin, and terminated its course beneath the integuments on the right buttock, over the ischiatic notch, from which place it was instantly extracted.

Immediately after being wounded, Captain S. was bled copiously, and the catheter was introduced to draw off the urine. This treatment afforded him a little relief. On the evening after the action, the army being ordered to retreat, he was conveyed to Fort George, a distance of seventeen miles, in an open waggon, during the inclemency of the night, the motion of which gave him considerable uneasiness. From thence he was carried on board a small vessel, conveyed across Lake Ontario, and landed at York on the evening of the fourth night after he received his injury, when, for the first time, he was committed to my care. When I entered his chamber, I found him stretched upon a hair mattress, his countenance pale and anxious, pulse quick, respiration hurried, and the urine, tinged with blood, flowing freely from the groin. The wound in the buttock, from which the ball had been extracted by a simple incision, looked clean and healthy. On the contrary, the wound in the groin appeared sloughy and inflamed, and the discharge from it was fetid and offensive. He made frequent attempts to void his urine by the natural passage, but, after the most painful and convulsive efforts, he could not effect it. He requested of me to introduce the catheter, for he had obtained considerable relief by that operation at a former period. On attempting to pass it, he felt severe spasms in the perinæum, and I was obliged to desist, having had the mortification of adding much to his sufferings by my interference. I directed an enema to be given, and an opiate to be taken at bed-time. On the following morning, when I visited him, a longitudinal piece of bone, nearly an inch in length, and about the thickness of a crow's quill, presented itself at the urethra, which I immediately extracted. He had spent a restless night; but the expulsion of this foreign body gave him a good deal of relief. For three weeks following he appeared nearly in the same state, only the wound in the groin looked more clean and healthy, from having its slough detached. However, he could now pass a few drops of urine by the natural passage, but in accomplishing this he felt most severe pain. The discharge of pus and urine was also copious, and extremely fetid, so that it was difficult to remain in the apartment for any length of time. The posterior wound now began to to inflame and suppurate. A piece of cloth was discharged, and the urine flowed through both openings. At this time also another piece of bone was passed by the urethra, which, when pressed between the fingers, mouldered down like a piece of earthy matter. About a fortnight after this period, the season became extremely hot, and the wound in the groin was over-run with maggots, which, for a short time, increased the suffering of my patient much, and really seemed to concentrate in one individual the highest degree of human misery. They were, however, soon destroyed by an injection of brandy and water. After this period, the wounds began to contract, and to be filled up with healthy granulations, while the functions of the bladder gradually resumed their office. The final closure of the wounds was much retarded by frequent attacks of intermittent fever, which indeed had proved fatal to a number of the wounded in hospital. Fowler's solution of arsenic was administered, as it was found most efficacious in putting a termination to the paroxysms of the fever. After having suffered much by three or four relapses of fever, my patient was ordered to the lower province of Canada, for a change of air and climate. He accordingly sailed on the 24th November from York, with his wounds completely closed, a period about three months and a half after they were first inflicted. On his arrival in the lower province, he was attacked with fever in the continued form, which was also attended with catarrhal symptoms. From the exertion of coughing, the wound in the groin was forced open, and the urine was again discharged. The fever abated in the space of eight or nine days; and in six weeks afterwards the wound in the groin was finally healed.

*Remarks.*—The wound, from its particular situation, excited a great deal of medical regard, for every surgeon who saw Captain S. despaired of his recovery. This case being complicated with splinters of bone, was one evidently not suited to the introduction of the elastic gum catheter, which has been found so useful in wounds of the bladder. The longitudinal piece of bone appeared to be a splinter from the anterior brim of the pelvis, and it was surely most surprising how it could find its way into a passage so narrow as the urethra. The exit of urine by the posterior wound had been prevented at first by the piece of pantaloon, which was afterwards discharged by suppuration.

Captain S. is now in England, and, since my arrival from America, I have had an opportunity of seeing him. The present statement with regard to his wound is as follows:—

The left limb is smaller than the right, and appears shorter when he walks or stands erect. He can walk a distance of three miles without resting, but is not able to ride on horse-back, from a tingling sensation, like the pricking of pins, in the perinæum, and which extends itself down the inside of the left leg. When he voids his urine, he stoops a good deal forward, presses in the abdominal muscles with his hands, in order to facilitate its expulsion. He feels at times a pain in his groin, especially in cold or damp weather,

 and and complains of much debility about the lumbar region. His bladder is not irritable, for he can contain his urine almost for any length of time. His urine, upon standing, deposits a thick sediment of a lateritious colour, but there is no appearance of pus in it. He has become rather corpulent, probably owing to his not being able to take his usual exercise.—*Edin. Jour.*

---

Mr. J. ACTON has communicated the following to the Editor of the *Philosophical Magazine:*—A friend of mine has lately presented me with two specimens of calcareous matter, taken from the bladders of two of his horses after they had died from disease,—one weighing nearly ten pounds, in an irregular form,—the other weighing about ten pounds and a quarter, of a conic form. As soon as I can possibly find time for their minute examination, it is my intention to send you the particulars.

---

Our readers will recollect the annular saw we some time ago announced, the invention of Mr. MACHELL, of Great Rider-street, St. James's. That valuable instrument has since undergone various improvements, which it was our intention to have described in the present Number. But, with all its advantages, it is supposed to be capable of others. These are now under trial, and, as soon as they are confirmed, we shall offer a full description of the instrument in its complete form.

---

Every friend of philosophy will, with us, regret the loss of Dr. WELLS, of Sergeants-inn, Fleet-street. For our next Number, we shall endeavour to procure a short biography of that truly philosophical physician.

We have also to lament the loss of Sir JAMES EARLE, the Master of the College of Surgeons, son-in-law to the celebrated Percival Pott, and, for several years, Senior Surgeon of St. Bartholomew's.

---

Mr. THOMAS BELL has been appointed to succeed the late Mr. FOX, as Lecturer on the Structure and Diseases of the Teeth, at Guy's Hospital.

---

Mr. T. J. PETTIGREW, F.L.S. Surgeon-extraordinary to their Royal Highnesses the Dukes of Kent and Sussex, will commence his Winter Course of Lectures on Anatomy, Physiology, and Pathology, on Friday the 17th of October, at eight o'clock in the evening precisely. The lectures will be continued every succeeding Wednesday and Friday, at the same hour, until completed.

Dr. MARCET intends to give a Course of Clinical Lectures, at Guy's Hospital, during the next winter.

Mr. GUTHRIE, Deputy-inspector of Military Hospitals, will commence his Autumn Course of Lectures on Surgery, on Monday, the 6th of October, at five minutes past eight in the evening, in the Waiting Room of the Royal Westminster Infirmary for Diseases of the Eye, Mary-le-bone Street, Piccadilly. To be continued on Mondays, Wednesdays, and Fridays.

*To the Editors of the London Medical and Physical Journal.*

GENTLEMEN,

By some accident, the account of our excursions in July was delayed too long for your last Number. In my next, besides that for September, you will receive a comparative statement of the periods for flowering and ripening of the seeds of several plants. This will be interesting to your botanical readers, and particularly to those at a distance from the metropolis.

The increase of my class, and the necessity imposed on the students in physic to acquire some knowledge of botany, has induced me to undertake a Winter Course of Reading and Conversation on Botanical Subjects. This, with a well-furnished library, and the occasional exhibition of dried or green-house specimens, will enable us to conclude the Summer or Spring Course much earlier. A further account of the plan will be prepared for your next Number.

I remain, gentlemen, yours, &c.

W. Salisbury.

### Mr. Salisbury's *Botanical Excursions for July.*

July 4, 1817. Battersea-fields and gardens in the neighbourhood.

Scabiosa Columbaria
Samolus Valerandi
Leucojum æstivum
Ægopodium Podagraria
Iris fœtida
Berberis vulgaris
Veronica spicata (gardens)
Vicia Cracca
Scirpus palustris
Polygonum aviculare
——— Hydropiper
Iris Pseudacorus
Scabiosa arvensis
——— nigra
Sparganium ramosum
Cynosurus cristatus
Acorus Calamus
Lapsana communis
Aira cœspitosa
Vicia sativa
Centaurea Cyanus
Leontodon hispidum
Alisma Plantago
Rumex obtusifolius
Thlaspi campestre
Triglochin palustre
Carex Pseudo Cyperus
Œnanthe crocata
Sedum Anglicum
Cratægus torminalis
Festuca fluitans
Bromus squarrosa
Geranium dissectum
Potentilla anserina
——— reptans
Sagina apetala
Juncus glaucus

July 12th. Kensington-gardens, Bayswater, &c.

Patamogeton natans
Achillea Millefolium
Agrostis fascicularis
Hordeum pratense
Alopecurus agrestis
Cuscuta Europœa
Hieracium dubium
Echium vulgare
Plantago major
Rosa canina
Geranium pusillum
Arenaria rubra
Rumex palustris
Polygonum amphibium
Sisymbrium Nasturtium
Ligustrum vulgare
Holcus lanatus
Veronica officinalis
——— agrestis
——— arvensis
Fumaria lutea
Sison inundatum
Festuca ovina
Avena flavescens
Veronica Anagallis

July 18th. Battersea-fields and the neighbourhood.

Solanum Dulcamara

Galium Aparine

Hordeum pratense

Urtica dioica

Fustuca loliacea

Rhinanthus Crista galli

Senecio Jacobœa

Phleum nodosum

Alopecurus pratensis

Sambucus nigra

Prunella vulgaris

Lychnis flos Cuculi

Crepis biennis

Plantago lanceolata

Hypericum quadrangulare

Tilia Europœa

Fumaria officinalis

Lathyrus pratensis

Trifolium pratense

——— repens

Rubus fruticosus

Lolium perenne

Œnanthe fistulosa

Euphorbia Helioscopia

Bryonia alba

July 25th. Battersea-fields and Wandsworth-common.

Malva rotundifolia

Hyoseris minima

Agrostemma Githago

Peucedanum Silaus

Lemna trisulca

——— minor

Scutellaria galericulata

Hydrocharis Morsus Ranæ

Mercurialis annua

Anagallis arvensis

Polygonum Convolvulus

Chenopodium album

Sison Amomum

Galeopsis Tetrahit

Daucus Carota

Lithospermum arvense

Chironia Centaurium

Campanula rotundifolia

Agrimonia Eupatorium

August 6th. Highgate Archway.

Carduus nutans
Erica vulgaris
Anthemis Cotula
Brassica Napus
Hypericum perforatum
Poa cristata
Tamus communis
Carduus acanthoides
Lychnis dioica v. alba
Gnaphalium germanicum
Ononis fructicosa
Potentilla verna

August 12th. Wimbledon-common.

Trifolium filiforme

Teucrium Scorodonium

Peplis Portula

Juncus articulatus

Genista anglica

Radiola millegrana

Juncus uliginosus

Festuca decumbens

Rosa spinosissima

Rhamnus Catharticus

Epilobium villosum

——— palustre

Trifolium glomeratum

Hypericum quadrangulare

Galeopsis tetrahit

Scrophularia aquatica

——— nodosa

Lythrum Salicaria

Senecio sylvatica

Jasione montana

Inula dysenterica

——— pulicaria

Medicago lupulina

Gnaphalium rectum

Pedicularis sylvaticus

Leontodon autumnale

Valeriana officinalis

Fumaria claviculata

Chelidonium majus

Scirpus fluitans

Ceratophyllum demersum

Poa rigida

Tragopogon porrifolium

Cynoglossum officinale

Matricaria Parthenium.

### REPORT OF DISEASES.

The autumn, though on the whole healthy, has been more sickly than for some years past. The diseases, however, have been mild. Dysenteries and diarrhœas, if not met, in their early stage, with antiphlogistic remedies, according to their severity, have proved extremely tedious, and, in a very few instances, fatal. The typhus fever, in its true form, has been chiefly confined to the poor Irish, and those who have associated with them. Fever, however, has been sporadic among all classes. In those whose mode of life has been more generous, the early symptoms have been severe, and not relieved without the lancet and other antiphlogistic remedies. If these have been omitted, the disease has proved fatal at an early period, or convalescence has proved extremely tedious. There has been less delirium than usually attends that form of fever, but the senses have been much impaired, and the conceptions greatly weakened.

A case of phthiriasis, or morbus pediculosus, has occurred in an elderly woman, the wife of a man in a decent employment. Though they sleep together, the husband has no difficulty in keeping himself clean; but it is the constant employment of the wife to destroy the vermin, yet they multiply faster than all her endeavours can prevent. They are the common body-louse; so that it is at least probable this disease is nothing more than a peculiar aptitude in the skin and juices of the subject to prove a nidus and food for this insect.

### A METEOROLOGICAL JOURNAL,

By Messrs. WILLIAM HARRIS and Co. 50, Holborn, London.

*From the 20th August to the 19th September, inclusive.*

| Day of Month. | Rain gauge | Therm | | | Barom | | De Luc's Hygrom. Dry | | Damp | | Wind. | | Atmospheric Variation. | | |
|---|---|---|---|---|---|---|---|---|---|---|---|---|---|---|---|
| August | | | | | | | | | | | | | | | |
| 20 | | 60 | 64 | 57 | $29^{6}$ | $29^{7}$ | | | 3 | 3 | SW | SW | Fine | | |
| 21 | ,05 | 59 | 65 | 54 | $29^{6}$ | $29^{6}$ | | | 3 | 3 | W | N | Fine | | |
| 22 | ,07 | 59 | 65 | 54 | $29^{6}$ | $29^{9}$ | | | 2 | 2 | NW | NW | Rain | Clo. | Rain |
| 23 | ,09 | 62 | 64 | 57 | $29^{9}$ | $29^{9}$ | | | 2 | 3 | N | NE | Fog | Fine | |
| 24 | | 59 | 62 | 55 | $29^{7}$ | $29^{4}$ | | | 4 | 4 | E | E | Rain | Clo. | Fine |
| 25 | ,06 | 57 | 60 | 54 | $29^{3}$ | $29^{2}$ | | | 4 | 5 | SE | S | Clo. | Rain | Fine |
| 26 | .08 | 57 | 60 | 55 | $29^{1}$ | $29^{1}$ | | | 5 | 5 | SSE | SSW | Clo. | Rain | Fine |
| 27 | ,12 | 57 | 63 | 57 | $29^{2}$ | $29^{5}$ | | | 5 | 6 | S | W | Rain | | Clo. |
| 28 | ,15 | 59 | 65 | 55 | $29^{6}$ | $29^{7}$ | | | 5 | 5 | SW | S | Fine | Rain | |
| 29 | ,08 | 59 | 66 | 56 | $29^{7}$ | $29^{7}$ | | | 4 | 4 | W | SW | Clo. | Rain | Fine |
| 30 | ,05 | 61 | 67 | 55 | $29^{9}$ | $29^{8}$ | | | 4 | 4 | SW | SSE | Fine | Clo. | Rain |
| 31 | ,03 | 59 | 69 | 53 | $29^{8}$ | $29^{9}$ | | | 4 | 3 | SW | SSW | Fine | | |
| Sept. | | | | | | | | | | | | | | | |
| 1 | | 59 | 65 | 51 | $29^{9}$ | $30^{1}$ | | | 3 | 3 | SW | S | Fine | | |
| 2 | | 57 | 67 | 59 | 30 | $29^{9}$ | | | 3 | 6 | ESE | E | Fine | | |
| 3 | | 62 | 72 | 60 | $29^{9}$ | $29^{9}$ | | | 6 | 6 | E | SE | Fine | | |
| 4 | | 63 | 70 | 58 | 30 | $30^{1}$ | | | 5 | 3 | W | W | Fine | | |
| 5 | | 61 | 69 | 58 | $30^{1}$ | $30^{1}$ | | | 1 | 1 | W | W | Fine | | |
| 6 | | 62 | 70 | 60 | 30 | $29^{9}$ | | 0 | 1 | 0 | S | SE | Fine | | |
| 7 | | 63 | 72 | 59 | $29^{8}$ | $29^{9}$ | 0 | 0 | 0 | 0 | NE | E | Fine | | |
| 8 | | 60 | 72 | 58 | $29^{9}$ | 30 | 3 | 2 | | | ENE | E | Fine | | |
| 9 | | 61 | 68 | 58 | 30 | 30 | | | 2 | 1 | N | N | Clo. | Fine | |
| 10 | ,14 | 60 | 69 | 57 | 30 | 30 | 0 | 0 | 0 | 0 | NE | N | Clo. | Fine | |
| 11 | | 61 | 67 | 55 | 30 | 30 | | | 2 | 2 | NE | SE | Clo. | | |
| 12 | | 63 | 66 | 57 | 30 | $29^{9}$ | | | 3 | 2 | W | W | Fog | Fine | |
| 13 | ,05 | 63 | 67 | 59 | $29^{9}$ | $29^{9}$ | | | 5 | 5 | NE | NE | Fine | | |
| 14 | | 64 | 68 | 57 | 30 | 30 | | | 5 | 5 | N | N | Fine | | |
| 15 | | 62 | 68 | 55 | 30 | 30 | | | 5 | 5 | NE | NE | Fine | | |
| 16 | | 60 | 65 | 53 | 30 | 30 | | | 10 | 12 | NE | NE | Fine | | |
| 17 | | 58 | 64 | 55 | $29^{9}$ | $29^{8}$ | | | 7 | 5 | N | N | Fine | | |
| 18 | ,05 | 59 | 65 | 57 | $29^{8}$ | $29^{8}$ | | | 5 | 5 | N | N | Fine | Rain | Fine |
| 19 | .08 | 58 | 62 | 55 | $29^{8}$ | $29^{9}$ | | | 3 | 2 | NE | E | Rain | | |

The quantity of rain fallen in the month of August is 1 inch and 94-100ths.

